# Ternary XBe_4_H_5_^−^ (X = Si, Ge, Sn, Pb) Clusters: Planar Tetracoordinate Si/Ge/Sn/Pb Species with 18 Valence Electrons

**DOI:** 10.3390/molecules28145583

**Published:** 2023-07-22

**Authors:** Yong-Xia Li, Li-Xia Bai, Jin-Chang Guo

**Affiliations:** 1 Department of Chemistry, Xinzhou Teachers University, Xinzhou 034000, China; 2 Nanocluster Laboratory Institute, Molecular Science Shanxi University, Taiyuan 030006, China; bailixia2021@163.com

**Keywords:** planar tetracoordinate silicon, planar tetracoordinate germanium, 18-vanlence-electron counting, double aromaticity, stability

## Abstract

As one of the important probes of chemical bonding, planar tetracoordinate carbon (ptC) compounds have been receiving much attention. Compared with ptC clusters, the heavier planar tetracoordinate silicon, germanium, tin, lead (ptSi/Ge/Sn/Pb) systems are scarcer and more exotic. The 18-valence-electron (ve)-counting is one important guide, though not the only rule, for the design of planar tetra-, penta-coordinate carbon and silicon clusters. The 18ve ptSi/Ge system is very scarce and needs to be expanded. Based on the isoelectronic principle and bonding similarity between the Al atom and the BeH unit, inspired by the previously reported ptSi global minimum (GM) SiAl_4_^2−^, a series of ternary 18 ve XBe4H5^−^ (X = Si, Ge, Sn, Pb) clusters were predicted with the ptSi/Ge/Sn/Pb centers. Extensive density functional theory (DFT) global minimum searches and high-level CCSD(T) calculations performed herein indicated that these ptSi/Ge/Sn/Pb XBe_4_H_5_^−^ (X = Si, Ge, Sn, Pb) clusters were all true GMs on their potential energy surfaces. These GMs of XBe_4_H_5_^−^ (X = Si, Ge, Sn, Pb) species possessed the beautiful fan-shaped structures: XBe_4_ unit can be stabilized by three peripheries bridging H and two terminal H atoms. It should be noted that XBe_4_H_5_^−^ (X = Si, Ge, Sn, Pb) were the first ternary 18 ve ptSi/Ge/Sn/Pb species. The natural bond orbital (NBO), canonical molecular orbitals (CMOs) and adaptive natural densitpartitioning (AdNDP) analyses indicated that 18ve are ideal for these ptX clusters: delocalized one π and three σ bonds for the XBe_4_ core, three Be-H-Be 3c-2e and two Be-H σ bonds for the periphery. Additionally, 2π plus 6σ double aromaticity was found to be crucial for the stability of the ptX XBe_4_H_5_^−^ (X = Si, Ge, Sn, Pb) clusters. The simulated photoelectron spectra of XBe_4_H_5_^−^ (X = Si, Ge, Sn, Pb) clusters will provide theoretical basis for further experimental characterization.

## 1. Introduction

The unique structures, peculiar properties and potential application prospect of planar hypercoordinate carbon clusters aroused great interest of theoretical and experimental chemists in the past fifty years, since Monkhrost firstly put forward a hypothetical transition-state structure containing the planar tetracoordinate carbon (ptC) in 1968 [1]. Based on bonding analysis of hypothetical planar CH_4_, Hoffmann, Alder and Wilcox Jr. proposed some effective strategies to stabilize the ptC species in 1970 [2]. In 1976, Schleyer and coworkers predicted the first stable ptC species, 1,1-dilithiocyclopropane [3]. In 1977, the V_2_(2,6-dimethoxyphenyl)_4_ complex was synthesized and characterized experimentally by Cotton et al., which can be seen as the first ptC compound [4]. Since then, a variety of novel ptC, planar pentacoordinate and hexa-coordinate carbon (ppC and phC) clusters, molecules, complexes even to nanomaterials have been reported [5,6,7,8,9,10,11,12,13]. Among them, the most noteworthy are a series of pentatomic ptC species. In 1999–2000, Wang and Boldyrev observed the ptC CAl_4_^−^, NaAl_4_C^−^, CAl_3_Si^−^ and CAl_3_Ge^−^ by using the photoelectron spectroscopy (PES) in gas phase combined with theoretical calculation [14,15,16]. Interestingly, these pentatomic ptC species had 18 valence electrons (ve) (except for CAl_4_^−^). It was found that 18ve counting is crucial for these pentatomic ptC clusters [17].

Silicon, germanium, tin and lead are in the row below carbon on the periodic table. According to the atomic radius data provided by Pyykkö [18], the covalent radius of C is only 0.75 Å, while the covalent radius of Si, Ge, Sn, Pb are 1.16, 1.21, 1.40 and 1.44 Å, respectively. Although the radius of the atom is increasing, the bonding properties are still similar. Silicon and germanium are dominated by tetrahedral coordination in their pure crystals and compounds. Heavier Sn and Pb atoms have stronger metallicity properties and weaker conjugation. The chemistry of Si/Ge/Sn/Pb is dominated by their tendency to form tetracoordinate tetrahedral structures, just as the tetrahedral carbon. The thermodynamic characteristics of silicon, lead and other metals were investigated by Kolesnikov and coworkers, which can help us to understand the chemical properties of Si and Pb more deeply [19,20]. Thus, the concepts of ptC, ppC and phC are naturally extended to the corresponding Si/Ge/Sn/Pb species. Using the PES combined with the DFT computations, Wang and coworkers identified the global minima of MAl_4_^−^ (M = Si, Ge) anions in 2000, which can be seen as the first ptSi and ptGe species [21]. They also found that the neutral MAl_4_ (M = Si, Ge) with 16ve remain planar, unlike *T_d_* CAl_4_. The experimental PES characterization of MAl_4_^−^ (M = Si, Ge) anions greatly stimulated the interest of theoretical researchers in designing and predicting ptSi species. In 2004, Li et al. proposed a general pattern to incorporate planar tetra-, penta-, hexa-, hepta- and octa-coordinate silicons in *C*_2_*_v_* B*_n_*E_2_Si series (E = CH, BH, or Si; *n* = 2–5) and *D*_8*h*_ B_8_Si [22]. Stimulated by the proposal of aromatic hydrocopper Cu*_n_*H*_n_* (*n* = 4–6) [23], Li cleverly designed the ptSi SiM_4_Cl_4_ (M = Ni, Pd, Pt), ppSi M_5_H_5_Si (M = Ag, Au, Pd, Pt) and phSi SiCu_6_H_6_ [24,25,26]. Replacing two carbon units of C_60_ with one silicon atom, Liu designed the spherical C_58_Si cluster with a quasi-ptSi in 2008 [27]. It is a pity that most of these designed clusters above were just local minima on the potential energy surfaces, which are difficult to observe or characterize experimentally.

In order to preserve the ptSi/Ge structure in the gas phase for spectral experimental detection, the target system should preferably be the global minimum (GM). Note the abbreviation “GM” refers to a structure that is lowest in energy on the potential energy surface of a specific molecular system, which is routinely used in physical chemistry or cluster literature. DFT investigations identified that the most stable structure of silicon carbonyl complex Si(CO)_4_ contained the ptSi center [28]. The square ptSi SiIn_4_^2−^ with 18ve was predicted by Alexandrova in 2012, in which aromaticity and covalency achieve a very good balance [29]. Nine pentaatomic ptSi GM clusters including Li_3_SiAs^2−^, HSiY_3_(Y = Al/Ga), Ca_3_SiAl_2_, Mg_4_Si^2−^, C_2_Li_2_Si, Si_3_Y_2_ (Y = Li/Na/K) were predicted theoretically by Xu and Ding in 2015 [30]. It should be noted that they are all 14ve species. Due to the larger atomic radius and weaker π conjugation ability, the heavier main-group elements seem to be much more difficult to have a ptX (X = C, Si, Ge, Sn, Pb) GM than the first-row main-group elements. Our group designed the ptSi/Ge superalkali cations Si_3_Li_3_^+^, Ge_3_Li_3_^+^, Si_3_Cu_3_^+^, Ge_3_Cu_3_^+^, which built the link between the ptSi/Ge and superalkali [31,32]. In 2016, Ding reported a series of planar tetracoordinate group 14 atom species C_2_Si_2_X^q^ (X = C, Si, Ge, Sn, Pb; q = +1, 0, −1) [33]. In 2022, a class of ternary 14ve clusters, ptSi/Ge/Sn/Pb XB_2_Be_2_ (X = Si, Ge, Sn, Pb), were computationally predicted by Zhai [34]. It should be noted that the global minima of XMg_4_Y (X = Si, Ge; Y = In, Tl) and SiMg_3_In_2_ clusters were predicted by Cui and coworkers, which represent the first series of ppSi/Ge clusters [35]. Very recently, the bare and ligand protected planar hexacoordinate silicon (phSi) SiSb_3_M_3_^+^ (M = Ca, Sr, Ba) clusters were predicted [36]. In addition, Li and co-workers designed the silagraphene consisting of the ptSis [37]. On the basis of the theoretical design of ptSi clusters and their characterization by PES, the researchers began to try to synthesize ptSi species in condensed phase. Important progress was made in laboratory synthesis and characterization of ptSi compounds. In 2018, calix[4]pyrrole hydridosilicate was experimentally synthesized, which contains the ptSi center [38]. The tetrasilane 1,3-diiodotetra-silabicyclo[1.1.0]butane, containing two ptSi atoms, was successfully isolated in 2020 [39]. In 2021, Greb et al. experimentally achieved the ptSi(IV) compound [40]. Recently, a series of thermally stable ptSi [Tp′(CO)_2_MSiC(R_1_)C(R_2_)M(CO)_2_Tp′] (M = Mo, W; R_1_ = R_2_ = Me or R_1_ = H, R_2_ = SiMe_3_, Ph; Tp′ = κ3-N,N′,N″-hydridotris (3,5-dimethylpyrazolyl) borate) complexes were synthesized and characterized [41]. Very recently, Driess reported the synthesis and reactivity of a peculiar ptSi compound [42].

Currently, the ptSi/Ge/Sn/Pb GMs are still scarce, and we are eager to design more examples. To design the ptSi/Ge/Sn/Pb clusters, the match of geometric size is crucial, as well as the number of electrons in the system. Our series of studies showed that the main group metal beryllium with low electronegativity is clever in bonding. Similar to Al, Be is an excellent ligand to stabilize planar hypercoordinate carbon. Here, we designed the ptSi/Ge/Sn/Pb XBe_4_H_5_^−^ (X = Si, Ge, Sn, Pb) clusters, using the Be as the ligands and H as the auxiliary atoms. The unbiased isomer search and high-level calculation suggest that the XBe_4_H_5_^−^ (X = Si, Ge, Sn, Pb) are all true GMs on the potential energy surfaces. We performed a DFT investigation on the structures, bonding characters and aromaticity of the XBe_4_H_5_^−^ (X = Si, Ge, Sn, Pb) clusters. To the best of our knowledge, there have been no theoretical or experimental investigations reported to date on the XBe_4_H_5_^−^ (X = Si, Ge, Sn, Pb) clusters. The ternary XBe_4_H_5_^−^ (X = Si, Ge, Sn, Pb) clusters in this work will further enrich the ptSi/Ge/Sn/Pb family and provide new ideas for researcher to carry out relevant theoretical design.

## 2. Computational Details

The extensive global minimum (GM) structural searches for XBe_4_H_5_^−^ (X = Si, Ge, Sn, Pb) clusters were performed at the PBE0/def2-SVP level using the Coalescence Kick (CK) program [43,44]. The current CK program was compiled by our research group. For each of XBe_4_H_5_^−^ (X = Si, Ge, Sn, Pb) clusters, around 4000 initial input structures (2000 singlets and 2000 triplets) were generated. The candidate low-lying structures were then reoptimized at the PBE0-D3(BJ)/def2-TZVPP level [45,46]. Frequency analyses were carried out at the same level to ensure that the reported structures are true minima. To obtain an accurate stability ordering, single point energy calculations were performed for the global minima and three lowest-energy isomers at the CCSD(T)/def2-TZVPP//PBE0-D3(BJ)/def2-TZVPP level [47]. The ultimate relative total energies of isomers were determined by the CCSD(T)/def2-TZVPP energies with the PBE0-D3(BJ)/def2-TZVPP zero-point energy corrections.

Natural bond orbital (NBO) [48] analyses were performed to get the Wiber bond indices (WBIs) and natural population analysis (NPA) charges. Here, we used the NBO 6.0 program, which was developed by Glendening, Badenhoop and coworkers in the University of Wisconsin [49]. To assess the aromatic character, the nucleus independent chemical shifts (NICSs) [50] were calculated for the GMs of XBe_4_H_5_^−^ (X = Si, Ge, Sn, Pb) clusters. Vertical detachment energies (VDEs) were calculated with the time-dependent PBE0 (TD-PBE0) method. All calculations for electric structures in this work were performed using the Gaussian 16 package [51]. Gaussian is a powerful commercial quantum chemistry package from Gaussian Inc. in USA, which was developed by Frisch and coworkers. The compositions of canonical molecular orbitals (CMOs) and adaptive natural density partitioning (AdNDP) [52] analyses were performed using the Multiwfn program [53]. Multiwfn program was developed by Lu at the Beijing Kein Research Center for Natural Sciences, which is free, open-source, high-efficient, very user-friendly and flexible. Molecular structures, CMOs and AdNDP bonding patterns were visualized using the CYLview [54], GaussView and Molekel [55] programs, respectively. CYLview was created by Legault in Université de Sherbrooke in 2009, which is a powerful and free graphics software. Molekel is also a free graphic package, which was developed by Varetto in Swiss National Supercomputing Centre.

## 3. Result and Discussion

### 3.1. Geometies and Stability

**Figure 1:**
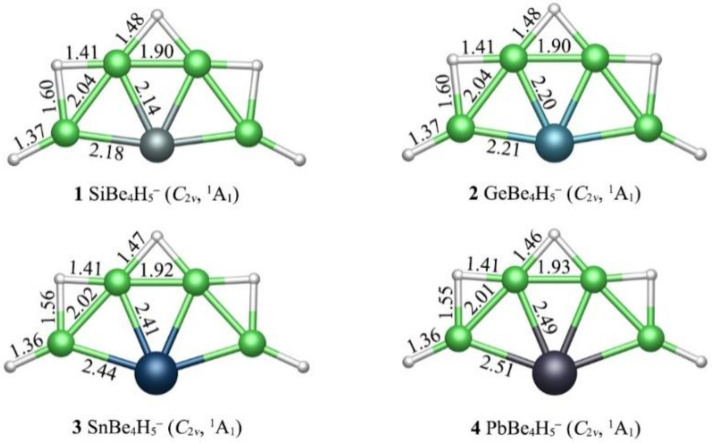
Optimized global minimum (GM) structures of the XBe_4_H_5_^−^ (X = Si, Ge, Sn, Pb) cluster at PBE0-D3(BJ)/def2-TZVPP level. The bond distances are presented (in Å).

The current study was primarily motivated by the previously reported 18ve ptC CAl_4_^2−^ [15] and ptSi SiAl_4_^2−^ [29]. Using the isoelectronic BeH units to replace the Al atoms of ptSi SiAl_4_^2−^ cluster and replace an electron with one H atom at the same time, the 18ve ptSi SiBe_4_H_5_^−^ cluster was formed. Previous studies showed that the bridge H has an obvious advantage over the terminal H in the ternary CBe_5_H_n_^n–4^ (*n* = 2–5) species [56]. The optimized geometries of GM structures **1**–**4** are depicted in Figure 1, along with their bond distances. The GMs of XBe_4_H_5_^−^ (X = Si, Ge, Sn, Pb) clusters possessed the fan-shaped XBe_4_ core, three bridging H and two terminal H atoms. The X center was tetracoordinated in plane by four Be atoms, whereas the H atoms were situated on the periphery and interacted only with Be atoms.

**Figure 2:**
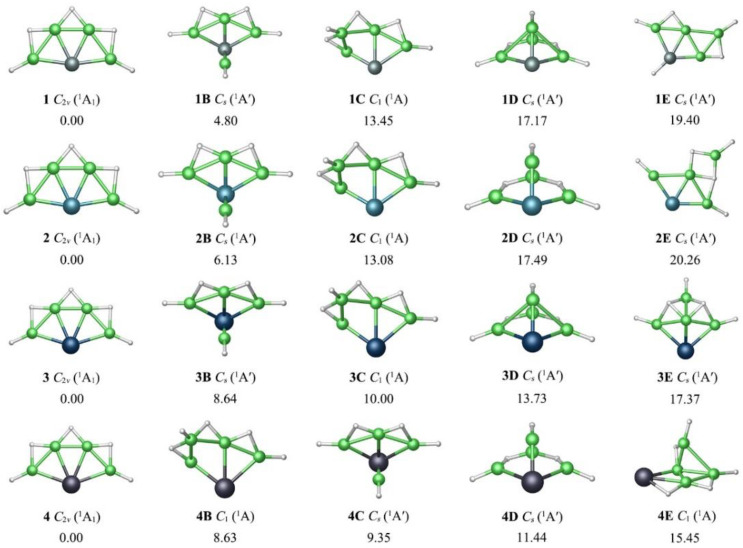
Optimized global-minimum structures **1**, **2**, **3**, **4** of XBe_4_H_5_^−^ (X = Si, Ge, Sn, Pb) clusters and their four low-lying isomers (***n*B**–***n*E**) at the PBE0-D3(BJ)/def2-TZVPP level. Relative energies are listed in kcal mol^−1^ at the single-point CCSD(T)/def2-TZVPP//PBE0-D3(BJ)/def2-TZVPP level, with zero-point energy (ZPE) corrections at PBE0-D3(BJ).

All GM clusters **1**–**4** assumed perfectly planar geometries with C_2v_ symmetry. The low-lying isomeric **nB**–**nE** structures are presented in Figure 2, along with the relative energies at single-point CCSD(T)//PBE0-D3(BJ)/def2-TZVPP level. Their optimized Cartesian coordinates for GMs **1**–**4** and the low-lying isomers **1B**–**4E** are provided in Table S1. C_2v_ XBe_4_H_5_^−^ (X = Si, Ge, Sn, Pb) (**1**–**4**) were the true GMs, being reasonably well separated from alternative structures by at least 4.80/6.13/8.64/8.63 kcal mol^−1^ at the single-point CCSD(T)/def2-TZVPP//PBE0-D3(BJ)/def2-TZVPP level. Rotating the Be-H at the end of **1**/**2**/**3/4** upward towards the plane, the **1B**/**2B**/**3B/4C** isomers with the C_s_ symmetry were obtained. In these structures, the X atoms formed the three-dimensional tetracoordination. Moving the terminal Be-H in **1**/**2**/**3/4** to the middle of adjacent two Be atoms and bonding with them as a bridging group, the isomers **1C**/**2C**/**3C/4B** can be obtained. In terms of energies, the **1**–**4** structures were the true GMs.

**Figure 3:**
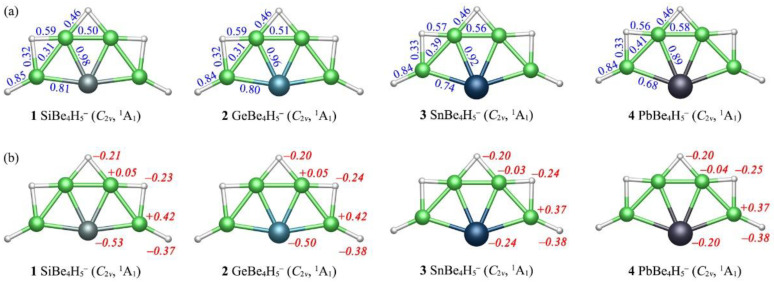
(**a**) Wiberg bond indices (WBIs, in blue color), and (**b**) atomic natural population analysis (NPA) charges (q, |e|, in red color) for **1**, **2**, **3**, **4**.

Although the X-Be/Be-H bonding in clusters **1**–**4** was partially polar in nature, owing to different electronegativities of the elements (Si: 1.90; Ge: 2.01; Sn: 1.96; Pb: 2.33; Be: 1.57; H: 2.20), the covalent radii [18] gave the upper bound of single X-Be, Be-H bonds to be 2.18/2.23/2.42/2.46 Å and 1.34 Å, respectively. The Be_4_ component in **1**–**4** had a curved chain shape. The Be–X distance was mainly affected by the bonding situation of Be atom. The Be atoms in **1**–**4** can be divided into two classes: one was bonding with two bridge H atom and the other was bonding with a bridge H and a terminal H. The Be-Si distances in **1** were in the range of 2.14–2.18 Å. The Si-Be links in **1** showed quite strong bonding, although their bond distances were uneven. The Be-H bond distances were in the range of 1.37–1.60 Å in **1**, while the Be–Be bond distances were in the range of 1.90–2.04 Å. As the radius of the X atom increased, the Be–X bond distances increased accordingly. The change in the X atom did not seem to affect the peripheral Be–Be, Be–H bond distances. As shown in Figure 3, the bond distances of Be–X, Be–H and Be–Be bonds seemed to be positively (albeit not strictly) correlated with their WBIs. The WBIs of Si–Be links in **1** were 0.81–0.98, indicating that there were effectively single bonds. As the electronegativity of X atom decreased gradually from Si to Pb, the WBIs of X-Be bonds also decreased to some extent. The Be–Be bonding in **1**–**4** was close to half bond, with the WBIs 0.31–0.58. The WBIs were 0.84–0.85, indicating that there was a single bond between terminal H and Be atom in **1**–**4**. The WBIs 0.32–0.59 were relatively small, suggesting there was only half bond between the bridging H and Be. These values did not correlate positively or precisely with bond distances, suggesting that the Be element is a flexible ligand for a ptSi/Ge/Sn/Pb configuration.

As shown in Figure 3, the ptSi/Ge/Sn/Pb centers in **1**–**4** possessed the negative charges −0.53/−0.50/−0.24/−0.20 |e|. The outer H atoms can also affect the charges on Be. The Be atoms bonding to two bridging H were nearly neutral, with only a small negative or positive charge. The Be atoms at both ends had the positive charges +0.37~+0.42 |e| in **1**–**4**. Since H was more electronegative than Be, both the bridging H and the terminal H carried a certain amount of negative charge (−20~−0.38 |e|). In short, the outer H ligands had a rather limited effect on chemical bonding in the XBe_4_ core.

The HOMO-LUMO energy gaps were computed at the PBE0-D3(BJ)-def2-TZVPP level to investigate the stability of the ptSi/Ge/Sn/Pb GM species. *C*_2*v*_ XBe_4_H_5_^−^ (X = Si, Ge, Sn, Pb) (**1**–**4**) had sizable HOMO-LUMO gaps (3.89, 3.85, 3.51 and 3.39 eV), suggesting that these ptX species were electronically robust. The large energy gap also indicates that a 18ve is all ideal for such ptSi/Ge/Sn/Pb clusters. 

### 3.2. Chemical Bonding

**Figure 4:**
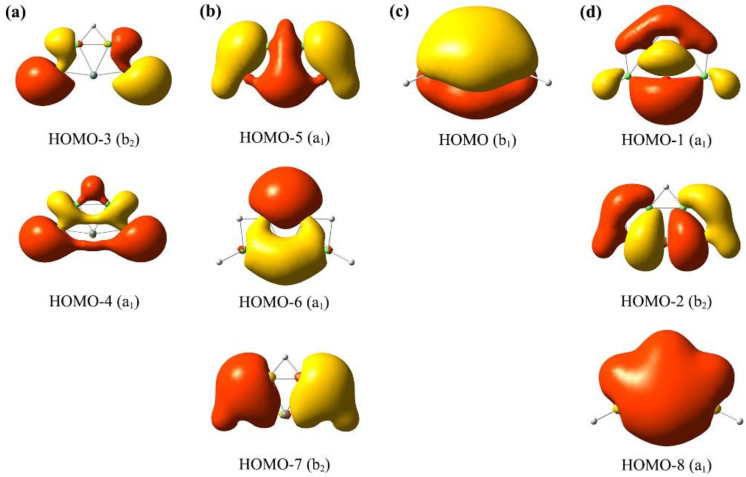
Analysis of canonical molecular orbitals (CMOs) of *C*_2*v*_ SiBe_4_H_5_^−^ (**1**) cluster. (**a**) Two σ CMOs for Lewis-type two-center two-electron (2c-2e) terminal B-H single σ bonds. (**b**) Three σ CMOs for delocalized Be-H-Be 3c-2e bonds. (**c**) One delocalized π CMO. (**d**) Three delocalized σ CMOs.

Why were these ptSi XBe_4_H_5_^−^ (X = Si, Ge, Sn, Pb) clusters stable? Canonical molecular orbitals (CMOs) analysis is important to help us understand the good stability of these ptSi/Ge/Sn/Pb GM clusters. The CMOs are fundamental in understanding the bonding nature in a molecular system. For the sake of simplicity, we took XBe_4_H_5_^−^ (**1**) as an example to reveal the bonding characteristics of XBe_4_H_5_^−^ (X = Si, Ge, Sn, Pb) clusters by molecular orbital analysis (Figure 4), aided with orbital composition analysis (Table S2). As shown in Figure 4, its nine occupied CMOs can be divided into four subsets, according to their compositions. Subset (a) had two CMOs: HOMO-3 and HOMO-4, which were primarily composed of Be 2s/2p and H 1s atomic orbitals (AOs). They can be transformed to two two-center two-electron (2c-2e) Be-H σ single bonds. Subset (b) had three CMOs (including HOMO-5, HOMO-6 and HOMO-7), corresponding to delocalized three-center two-electron (3c-2e) Be–H–Be bonds. Subset (c) was the π framework on SiBe_4_ unit, which involved only the HOMO. The 2π electrons were contributed by SiBe_4_ core, which endowed the 2π aromaticity according to the (4*n* + 2) Hückel rule. Lastly, subset (d) included HOMO-1, HOMO-2 and HOMO-8, which was a 6σ system with major contributions from Be 2s/2p and Si 3s/3p atomic orbitals (AOs). It is stressed that both π and σ frameworks were truly delocalized over the SiBe_4_ core; they could not be transformed to Lewis-type π/σ bonds. Thus, one π CMO (c) and three delocalized σ CMOs (d) suggest that there was two-fold delocalization in *C*_2*v*_ SiBe_4_H_5_^−^ (**1**), that is, double (2π and 6σ) aromaticity.

**Figure 5:**
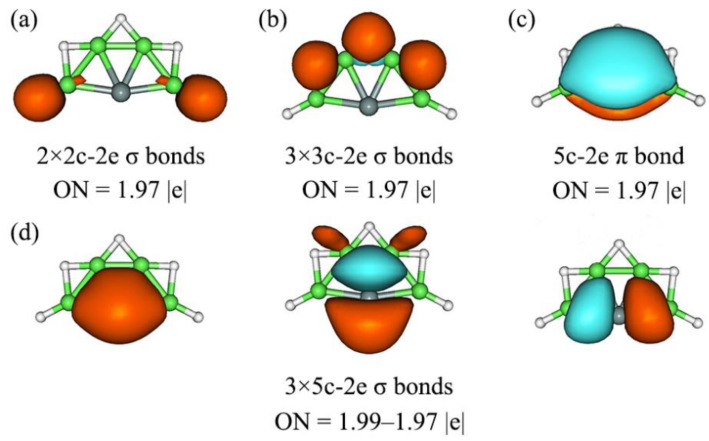
Chemical bonding pattern for SiBe_4_H_5_^−^ (**1**) cluster, according to the adaptive natural density partitioning (AdNDP) analysis. Occupation numbers (ONs) are shown. (**a**) two localized Be–H σ bonds; (**b**) three Be–H–Be 3c-2e σ bonds; (**c**) one delocalized π bond; (**d**) three delocalized σ bonds.

To gain insight into the bonding nature of **1**–**4**, we performed the AdNDP analyses. AdNDP analysis is an effective tool developed by Zubarev and Boldyrev for revealing the chemical bonding nature of one molecular. As an extension of the NBO analysis, AdNDP is simpler and more intuitive than the CMOs. The AdNDP analysis, thus, recovers not only the classical Lewis bonding elements (such as lone-pairs and 2c-2e bonds) but also nonclassical, delocalized nc-2e bonds. Here, we still took **1** as an example to reveal the bonding nature of these clusters through AdNDP analysis. The AdNDP scheme for SiBe_4_H_5_^−^ (**1**) is illustrated in Figure 5. SiBe_4_H_5_^−^ (**1**) was a 18 ve system. As shown in Figure 5, there were two Be–H 2c-2e localized σ bonds and three 3c-2e Be–H–Be σ bonds in **1**, with the idea ON 1.97|e|. These ten electrons were for peripheral σ bonding, which were irrelevant to the Si center. There were delocalized (5c-2e) one π and three σ bonds on the SiBe_4_ cores in **1**, with the idea occupation number (ON) from 1.97 to 1.99 |e|. The delocalized π/σ bonding around Si center involved eight electrons only, which confirmed the bonding pattern from CMOs analysis. Therefore, these four orbitals also offered 2π and 6σ double aromaticity according to the Hückel 4*n* + 2 rule, which was beneficial to stabilize the ptSi structure. The CMOs and AdNDP bonding pattern of XBe_4_H_5_^−^ (X = Ge, Sn, Pb) was similar to that of SiBe_4_H_5_^−^. 

### 3.3. Double Aromaticity

**Figure 6:**
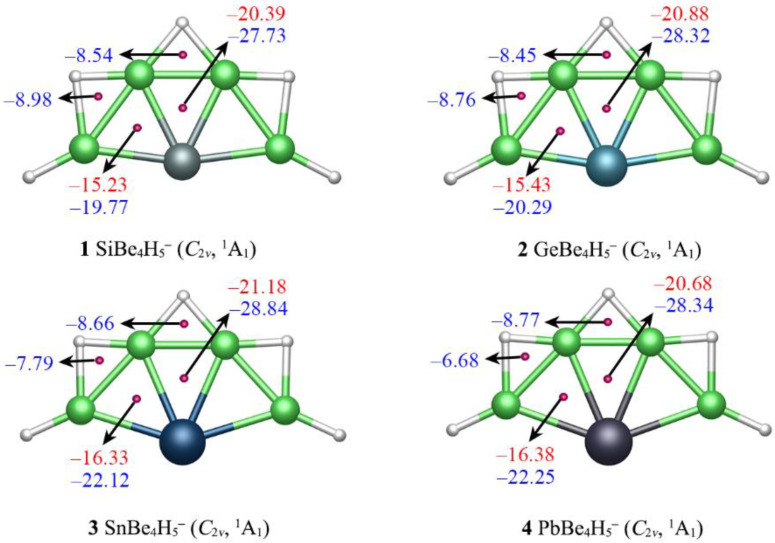
Nucleus independent chemical shifts (NICSs) for clusters **1**–**4**. NICS (0), shown in blue, is calculated at the center of a triangle. NICS (1), shown in red, is calculated at 1 Å above the center of a triangle.

The nucleus-independent chemical shift (NICS) calculations were performed to quantitatively characterize the aromaticity of XBe_4_H_5_^−^ (X = Si, Ge, Sn, Pb) clusters. NICS(0) and NICS(1) were calculated at the Be–X–Be, Be–H–Be triangles centers and at 1 Å above them, which can semi-quantitatively characterize the σ and π aromaticity, respectively. As shown in Figure 6, the NICS(0) values at the geometric center of Be–Si–Be triangles were −27.73/−19.77 ppm, suggesting that SiBe_4_H_5_^−^ possessed good σ aromaticity. Accordingly, the NICS(1) values of Be–Si–Be triangles were −20.39/−15.23 ppm, indicating that SiBe_4_H_5_^−^ also possessed good π aromaticity. In addition, the NICS(0) of Be–H–Be triangles was highly negative, revealing that the σ aromatic characteristics were present in these regions. The situations of XBe_4_H_5_^−^ (X = Ge, Sn, Pb) (**2**–**4**) were similar to those of SiBe_4_H_5_^−^ (**1**). Thus, **1**–**4** had both π and σ double aromaticity, which is consistent with the conclusion obtained from the AdNDP analysis. Boldyrev and Simons described in an early paper that one π and three σ bonds around C center are crucial for an 18ve ptC cluster. Similar to ptC clusters, one π and three σ bonds are also important for ptSi species, although total electrons of system do not need to obey the 18ve counting.

**Figure 7:**
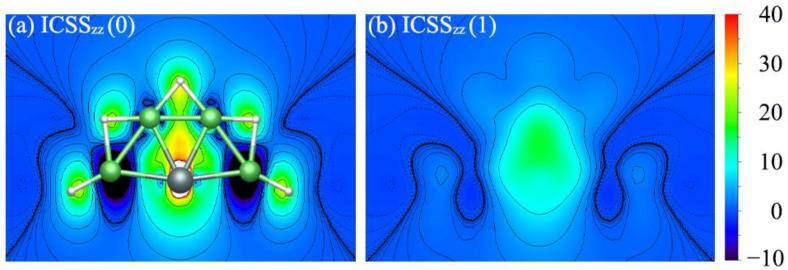
Color-filled maps of (**a**) ICSS(0)zz and (**b**) ICSS(1)zz (in ppm) for the SiBe_4_H_5_^−^ (**1**) cluster. Positive values indicate aromaticity. 0 and 1 in parentheses represent the height above the molecular planes (in Å).

However, it seems a little inadequate to reveal the aromaticity of the system through the NICS values of a few points. The magnetic criterion isochemical shielding surface (ICSS) [57] calculation was handled in a three-dimensional grid of lattice points and direction and anisotropy effects can be quantified in a more straightforward way. To more intuitively observe the aromaticity, the color-filled maps of ICSS_zz_ (0) and ICSS_zz_ (1) are shown in Figure 7. Note here that positive ICSS_zz_ values indicate diatropic ring currents and aromaticity. Similarly, Figure 7a,b indicate that SiBe_4_H_5_^−^ had σ and π double aromaticity. The situation of XBe_4_H_5_^−^ (X = Ge, Sn, Pb) (**2**–**4**) was similar to that of SiBe_4_H_5_^−^ (**1**).

### 3.4. Simulated Photoelectron Spectra

**Figure 8:**
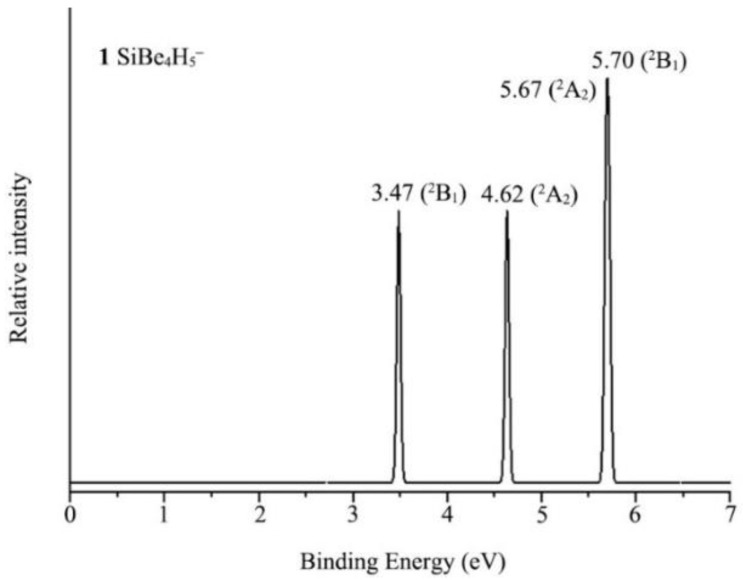
Simulated photoelectron spectra of SiBe_4_H_5_ cluster at the time-dependent PBE0/def2-TZVPP (TD-PBE0) level. The labeled electronic transitions are the ground state and excited states of the corresponding neutral SiBe_4_H_5_ cluster.

Given the fact that ptSi/Ge/Sn/Pb clusters are scarce in chemistry, we shall compute their electronic properties. Anion XBe_4_H_5_^−^ (X = Si, Ge, Sn, Pb) (**1**–**4**) clusters were reasonably well-defined on their potential energy surfaces. Photoelectron spectroscopy (PES) is a powerful tool to characterize anion clusters in the gas phase. To aid future experimental characterizations of the XBe_4_H_5_^−^ (X = Si, Ge, Sn, Pb) clusters, their PESs were simulated at the time-dependent PBE0/def2-TZVPP (TD-PBE0) level. The simulated PES spectrum of SiBe_4_H_5_^−^ (**1**) is presented in Figure 8, which had three well-separated bands in the 3–7 eV binding energy regime. The ground-state vertical detachment energy (VDE) of **1** was calculated to be 3.47 eV at the CCSD(T)/def2-TZVPP/PBE0-D3(BJ)/def2-TZVPP level, respectively, which is relatively high in binding energies. As shown in Figure S1, the simulated PESs of XBe_4_H_5_^−^ (X = Ge, Sn, Pb) (**2**–**4**) clusters were basically the same as SiBe_4_H_5_^−^ (**1**).

## 4. Conclusions

In conclusion, we computationally designed the XBe_4_H_5_^−^ (X = Si, Ge, Sn, Pb) clusters with ptSi/Ge/Sn/Pb centers, which were isoelectronic with the previously reported ptSi GM SiAl_4_^2−^. The ptSi/Ge/Sn/Pb GM clusters had the perfect fan-shaped XBe_4_ cores, which were protected by peripheral three bridging and two terminal H atoms. They were true global minima on the potential energy surfaces, through unbiased global structural searches and high-level quantitative calculations. The XBe_4_H_5_^−^ (X = Si, Ge, Sn, Pb) clusters represent the first ternary ptSi/Ge/Sn/Pb species with 18 ve. The NBO, CMOs and AdNDP analyses indicated that 18ve are ideal for these ptX clusters: delocalized one π and three σ bonds for the XBe_4_ core, three Be-H-Be 3c-2e and two Be-H σ bonds for the periphery. According to the Hückel 4*n* + 2 rule, the XBe_4_H_5_^−^ (X = Si, Ge, Sn, Pb) clusters possess 2π + 6σ double aromaticity, which underlies their planarity and stability. The current finding suggests further opportunities to design novel planar hypercoordinate Si/Ge/Sn/Pb species, via tuning the ligands, auxiliary atoms and altering the electron counting.

## Data Availability

All data reported in this study are available upon request by contact with the corresponding author.

## Supplementary Materials

The following supporting information can be downloaded at: https://www.mdpi.com/1420-3049/28/14/5583/s1, Figure S1: Simulated photoelectron spectra of XBe_4_H5^−^ (X = Ge, Sn, Pb) clusters at the time dependent PBE0/def2-TZVPP (TD-PBE0) level; Table S1: Cartesian coordinates for global minimum (GM) clusters minimum (GM) clusters 1–4 of the XBe4H5^−^(X = Si, Ge, Sn, Pb) series at the PBE0-D3(BJ)/def2-TZVPP level, along with four lowest-lying ***n*B**–***n*E** isomeric structures; Table S2: Orbital composition analysis of canonical molecular orbitals (CMOs of the global minimum structure 1 (C_2v_, ^1^A_1_ of SiBe_4_H5^−^ cluster.
