# Supplementary material for: Ternary XBe4H5− (X = Si, Ge, Sn, Pb) Clusters: Planar Tetracoordinate Si/Ge/Sn/Pb Species with 18 Valence Electrons
Source: Molecules. 2023 Jul 22;28(14):5583. doi: 10.3390/molecules28145583 (PMC10385292; doi:10.3390/molecules28145583)

SUPPLEMENTARY INFORMATION

Ternary $XBe_4H_5^-$ (X = Si, Ge, Sn, Pb) clusters：Planar Tetracoordinate Si/Ge/Sn/Pb species with 18 valence electrons

Yong-Xia Li[1,*], Li-Xia Bai[2] and Jin-Chang Guo[2,*]

[1]Department of Chemistry, Xinzhou Teachers University, Xinzhou 034000, China;

[2]Nanocluster Laboratory Institute of Molecular Science Shanxi University, Taiyuan, 030006, China;

*Correspondence: liyongxia0126@163.com (Y.-X.L.); guojc@sxu.edu.cn (J.-C.G.)

Table of Contents

Table S1. Cartesian coordinates for global-minimum (GM) clusters **1**–**4** of the $XBe_4H_5^-$ (X = Si, Ge, Sn, Pb) series at the PBE0-D3(BJ)/def2-TZVPP level, along with four lowest-lying ***n*B**–***n*E** isomeric structures.

Table S2. Orbital composition analysis of canonical molecular orbitals (CMOs) of the global-minimum structure **1** (C_{2v}, $^1A_1$) of $SiBe_4H_5^-$ cluster.

Figure S1. Simulated photoelectron spectra of $XBe_4H_5^-$ (X = Ge, Sn, Pb) clusters at the time-dependent PBE0/def2-TZVPP (TD-PBE0) level. The labeled electronic transitions are the ground state and ex-cited states of the corresponding neutral XBe_4H_5 (X = Ge, Sn, Pb) clusters.

Table S1. Cartesian coordinates for global-minimum (GM) clusters **1–4** of the $XBe_4H_5^-$ (X = Si, Ge, Sn, Pb) series at the PBE0-D3(BJ)/def2-TZVPP level, along with four lowest-lying ***n*B–*n*E** isomeric structures.

1

Be	2.16466100	0.39405000	0.00000000
Be	-2.16466100	0.39405000	0.00000000
Be	-0.94784100	-1.24187500	0.00000000
Be	0.94784100	-1.24187500	0.00000000
H	0.00000000	-2.38480500	0.00000000
H	3.37810900	1.02594500	0.00000000
H	-2.35573500	-1.19695200	0.00000000
H	-3.37810900	1.02594500	0.00000000
H	2.35573500	-1.19695200	0.00000000
Si	0.00000000	0.67924400	0.00000000

1B

Si	-0.91886400	-0.02391300	0.00000000
Be	1.00745600	0.93872100	0.00000000
Be	0.33101500	0.38804200	1.80876800
Be	0.33101500	-1.67748900	0.00000000
Be	0.33101500	0.38804200	-1.80876800
H	1.56799300	1.23826900	-1.27457100
H	0.35148100	0.28032200	-3.17314600
H	1.02314500	-2.85167700	0.00000000
H	1.56799300	1.23826900	1.27457100
H	0.35148100	0.28032200	3.17314600

1C

Si	0.54512700	-0.88594800	0.20280600
Be	-1.95795700	0.57903700	0.44277300
Be	-0.08688000	1.10805700	-0.34978700
Be	-1.15722000	-0.76971700	-0.84435800
Be	1.91485200	0.82763900	-0.00679400
H	-2.49941100	-0.37060000	-0.70060400
H	1.00986300	2.03291300	-0.23971900
H	-2.87885600	0.85626900	1.41989800
H	3.24980500	1.13303300	0.02821600
H	-1.36436700	1.77159400	-0.31441100

1D

Si	1.10662100	-0.01828000	0.00000000
Be	-0.31430300	0.46423700	1.68910300
Be	-1.50305400	0.12863300	0.00000000
Be	-0.31430300	-1.42984900	0.00000000
Be	-0.31430300	0.46423700	-1.68910300
H	-0.15931600	0.85801500	2.99566100
H	-1.81271100	0.71340900	1.26520000
H	-1.81271100	0.71340900	-1.26520000
H	-1.76479900	-1.39596300	0.00000000

H	-0.15931600	0.85801500	-2.99566100

1E

Si	-0.48114600	-1.02016100	0.00000000
Be	1.45385400	-0.35171300	0.00000000
Be	-1.97515600	0.64313200	0.00000000
Be	0.00000000	1.07112200	0.00000000
Be	2.17643200	1.48134900	0.00000000
H	3.04644400	2.53864100	0.00000000
H	2.83148100	0.03865100	0.00000000
H	-1.24558000	-2.30726700	0.00000000
H	-3.34116600	0.72604900	0.00000000
H	-1.17565200	1.91061200	0.00000000

2

Be	2.18551400	0.12240700	0.00000000
Be	-2.18551400	0.12240700	0.00000000
Be	-0.95219000	-1.50574700	0.00000000
Be	0.95219000	-1.50574700	0.00000000
H	0.00000000	-2.64149100	0.00000000
H	3.40415300	0.74260400	0.00000000
H	-2.35894200	-1.46955200	0.00000000
H	-3.40415300	0.74260400	0.00000000
H	2.35894200	-1.46955200	0.00000000
Ge	0.00000000	0.47381600	0.00000000

2B

Ge	-0.62816200	-0.02037800	0.00000000
Be	1.36372900	0.92701000	0.00000000
Be	0.67271500	0.42287600	1.82668300
Be	0.67271500	-1.68785200	0.00000000
Be	0.67271500	0.42287600	-1.82668300
H	1.92315000	1.23585100	-1.27276400
H	0.68697900	0.35465200	-3.19280200
H	1.35341300	-2.86855500	0.00000000
H	1.92315000	1.23585100	1.27276400
H	0.68697900	0.35465200	3.19280200

2C

Ge	0.54462900	-0.50075600	0.08705200
Be	-2.40086900	0.34811400	0.43641600
Be	-0.67917700	1.30852700	-0.33252000
Be	-1.26109100	-0.75830000	-0.84162800
Be	1.34369000	1.59202300	-0.03141100
H	-2.66233600	-0.72342400	-0.67487200
H	0.12370800	2.48887700	-0.16476300
H	-3.35074100	0.39606500	1.42211000
H	2.53444500	2.26608300	0.01629300
H	-2.08341300	1.63515100	-0.30786900

2D

Ge	0.76788100	0.00003100	-0.00812600
Be	-0.76377400	-1.68488500	-0.45278900
Be	-1.95472900	0.00008800	-0.05927100
Be	-0.76405900	1.68532000	-0.45201100
Be	-0.68395800	-0.00086900	1.43918600
H	-2.14378200	-0.00089000	1.45125300
H	-2.26836600	-1.26405900	-0.64282400
H	-0.61257700	2.97257000	-0.90389100
H	-0.61309300	-2.97244000	-0.90401700
H	-2.26830400	1.26522300	-0.64096200

2E

Ge	0.00000000	0.88446000	0.00000000
Be	0.21694300	-1.24713200	0.00000000
Be	-0.31723400	-3.18686800	0.00000000
Be	-1.74800100	-0.41207600	0.00000000
Be	1.99656100	0.16006800	0.00000000
H	-3.10109900	-0.58359300	0.00000000
H	0.96439900	-2.50725300	0.00000000
H	-0.66294700	-4.49315700	0.00000000
H	3.35037000	-0.00556700	0.00000000
H	-1.14379900	-1.96910700	0.00000000

3

Be	2.33431900	-0.27786100	0.00000000
Be	-2.33431900	-0.27786100	0.00000000
Be	-0.96039700	-1.76464900	0.00000000
Be	0.96039700	-1.76464900	0.00000000
H	0.00000000	-2.87620200	0.00000000
H	3.57395700	0.29156500	0.00000000
H	-2.36875900	-1.84005100	0.00000000
H	-3.57395700	0.29156500	0.00000000
H	2.36875900	-1.84005100	0.00000000
Sn	0.00000000	0.44626500	0.00000000

3B

Sn	-0.56477600	-0.00030100	-0.04325600
Be	1.74924100	0.00120300	-0.67809600
Be	1.09124500	1.88015500	-0.33189700
Be	0.95273100	-0.00010000	1.74198700
Be	1.09355000	-1.87868400	-0.33250900
H	2.41488100	-1.25070700	-0.87025000
H	1.13345500	-3.24430400	-0.31910100
H	1.60070500	-0.00004300	2.94202500
H	2.41332300	1.25401000	-0.86985700
H	1.12935500	3.24582000	-0.31797700

3C

Sn	0.57417800	-0.29583800	0.05115700
Be	-2.81829800	-0.13660300	0.43766500
Be	-1.29270100	1.20895400	-0.26352000
Be	-1.40849100	-0.89609400	-0.83298000
Be	0.53334700	2.16651100	-0.05532200

H	-2.77531400	-1.22691100	-0.64867300
H	-0.90995900	2.58083800	-0.07014800
H	-3.78531300	-0.26931200	1.39558400
H	1.43728200	3.19078400	-0.02061300
H	-2.73101300	1.14542400	-0.35734500

3D

Sn	-0.14419000	0.63971500	0.00000000
Be	-0.14419000	-1.24445800	1.74138100
Be	0.38455900	-2.28385600	0.00000000
Be	1.60468300	-0.72781700	0.00000000
Be	-0.14419000	-1.24445800	-1.74138100
H	-0.62462600	-1.18556300	3.02282300
H	-0.10941900	-2.72345000	1.26924300
H	-0.10941900	-2.72345000	-1.26924300
H	1.87413600	-2.16539300	0.00000000
H	-0.62462600	-1.18556300	-3.02282300

3E

Sn	-0.28923500	0.62597600	0.00000000
Be	0.43846100	-2.57208400	0.00000000
Be	1.46122300	-0.91234800	0.00000000
Be	0.43846100	-0.86931700	1.79363900
Be	0.43846100	-0.86931700	-1.79363900
H	0.23698400	-1.30194200	-3.07144000
H	1.45282800	-1.97827300	1.05197500
H	-0.02428000	-3.84612800	0.00000000
H	0.23698400	-1.30194200	3.07144000
H	1.45282800	-1.97827300	-1.05197500

4

Be	2.36321200	-0.52191000	0.00000000
Be	-2.36321200	-0.52191000	0.00000000
Be	-0.96389100	-1.96847800	0.00000000
Be	0.96389100	-1.96847800	0.00000000
H	0.00000000	-3.07056400	0.00000000
H	3.59696600	0.05914600	0.00000000
H	-2.37260300	-2.07617300	0.00000000
H	-3.59696600	0.05914600	0.00000000
H	2.37260300	-2.07617300	0.00000000
Pb	0.00000000	0.32960600	0.00000000

4B

Pb	0.43924000	-0.16236200	0.02986100
Be	-3.07182400	-0.37721600	0.43786800
Be	-1.67496300	1.12682300	-0.24566200
Be	-1.57080000	-0.96075800	-0.81555600
Be	-0.00271200	2.33333700	-0.06220200
H	-2.88607000	-1.47021200	-0.61896400
H	-1.48693900	2.53553200	-0.03332400
H	-4.04079900	-0.57330600	1.38258300
H	0.76164500	3.46484800	-0.01616000
H	-3.08431600	0.86806800	-0.42049800

4C

Pb	0.41391800	-0.00020100	-0.01676300
Be	-1.95688100	0.00166600	-0.71324700
Be	-1.34832700	-1.88946200	-0.35550000
Be	-1.32448900	-0.00095200	1.68226800
Be	-1.34575700	1.89160000	-0.35383800
H	-2.64493400	1.24694900	-0.89776700
H	-1.37982400	3.25757700	-0.37145000
H	-1.98309500	-0.00163100	2.87826600
H	-2.64673900	-1.24241500	-0.89877500
H	-1.38488800	-3.25536800	-0.37442200

4D

Pb	-0.09419700	0.46538500	0.00000000
Be	-0.09419700	-1.53611800	1.73843600
Be	0.40054300	-2.58942100	0.00000000
Be	1.61482700	-1.02877800	0.00000000
Be	-0.09419700	-1.53611800	-1.73843600
H	-0.62389000	-1.44852900	2.99876200
H	-0.10603400	-3.01405100	1.27115000
H	-0.10603400	-3.01405100	-1.27115000
H	1.87605500	-2.47468700	0.00000000
H	-0.62389000	-1.44852900	-2.99876200

4E

Be	1.63102000	-1.00088800	0.39673800
Be	2.09991800	1.35006800	0.56487200
Be	1.62338200	0.43092300	-1.02681100
Be	3.46251200	-0.59367900	-0.22100700
H	1.87435000	1.82125300	-0.86927500
H	2.55995600	2.18691400	1.54926100
H	2.85853500	-1.74609100	0.58755300
H	4.80971200	-0.66254900	-0.45433900
H	0.37865900	-1.65016700	0.78836200
Pb	-0.58229900	-0.00847600	-0.00557000

Table S2. Orbital composition analysis of canonical molecular orbitals (CMOs) of the global-minimum structure **1** (C_{2v}, $^1A_1$) of $SiBe_4H_5^-$ cluster.

CMO	Si (%)		Be_4 (%)		H_5 (%)
	s/p	total	s/p	total	s
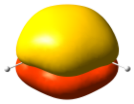 HOMO (b_1)	0.0/43.9	43.9	0.0/56.1	56.1	0.0
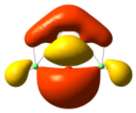 HOMO-1 (a_1)	9.3/50.4	59.7	1.2/27.5	28.7	10.0
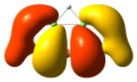 HOMO-2 (b_2)	0.0/49.3	49.3	2.5/34.7	37.2	15.6
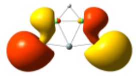 HOMO-3 (b_2)	0.0/1.0	1.0	12.6/20.3	32.9	66.1
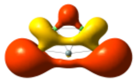 HOMO-4 (a_1)	0.0/3.4	3.4	11.0/21.1	32.1	64.5
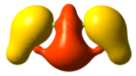 HOMO-5 (a_1)	12.2/0.0	12.2	4.8/31.0	35.8	51.6
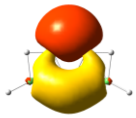 HOMO-6 (a_1)	10.3/0.7	11.0	10.0/41.9	51.9	45.7
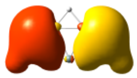 HOMO-7 (b_2)	0.0/7.8	7.8	22.0/22.7	44.7	47.4
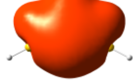 HOMO-8 (a_1)	32.7/3.7	36.4	31.4/15.8	47.2	15.3

Figure S1. Simulated photoelectron spectra of $XBe_4H_5^-$ (X = Ge, Sn, Pb) clusters at the time-dependent PBE0/def2-TZVPP (TD-PBE0) level. The labeled electronic transitions are the ground state and ex-cited states of the corresponding neutral XBe_4H_5 (X = Ge, Sn, Pb) clusters.

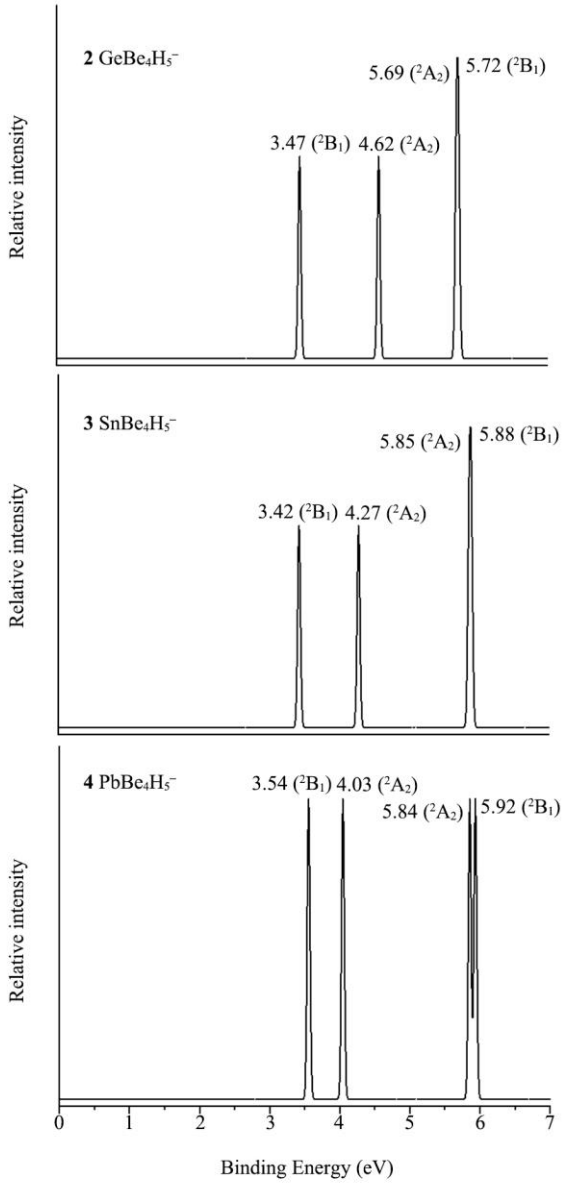

Supplement: Supplementary file 1 [file molecules-28-05583-s001.zip › molecules-2506677-supplementary.pdf]
